# Improved Hypertension Risk Assessment with Photoplethysmographic Recordings Combining Deep Learning and Calibration

**DOI:** 10.3390/bioengineering10121439

**Published:** 2023-12-18

**Authors:** Jesús Cano, Vicente Bertomeu-González, Lorenzo Fácila, Fernando Hornero, Raúl Alcaraz, José J. Rieta

**Affiliations:** 1 BioMIT.org, Electronic Engineering Department, Universitat Politecnica de Valencia, 46022 Valencia, Spain; jecaser@upv.es; 2 Cardiovascular Research Group, Clinical Medicine Department, Miguel Hernández University, 03202 Alicante, Spain; vbertomeu@umh.es; 3 Cardiology Department, General University Hospital Consortium of Valencia, 46014 Valencia, Spain; lfacila@gmail.com; 4 Cardiovascular Surgery Department, Hospital Clínico Universitario de Valencia, 46010 Valencia, Spain; hornero_fer@gva.es; 5 Research Group in Electronic, Biomedical and Telecommunication Engineering, University of Castilla-La Mancha, 16071 Cuenca, Spain; raul.alcaraz@uclm.es

**Keywords:** blood pressure, hypertension, photoplethysmography, calibration, deep learning

## Abstract

Hypertension, a primary risk factor for various cardiovascular diseases, is a global health concern. Early identification and effective management of hypertensive individuals are vital for reducing associated health risks. This study explores the potential of deep learning (DL) techniques, specifically GoogLeNet, ResNet-18, and ResNet-50, for discriminating between normotensive (NTS) and hypertensive (HTS) individuals using photoplethysmographic (PPG) recordings. The research assesses the impact of calibration at different time intervals between measurements, considering intervals less than 1 h, 1–6 h, 6–24 h, and over 24 h. Results indicate that calibration is most effective when measurements are closely spaced, with an accuracy exceeding 90% in all the DL strategies tested. For calibration intervals below 1 h, ResNet-18 achieved the highest accuracy (93.32%), sensitivity (84.09%), specificity (97.30%), and F1-score (88.36%). As the time interval between calibration and test measurements increased, classification performance gradually declined. For intervals exceeding 6 h, accuracy dropped below 81% but with all models maintaining accuracy above 71% even for intervals above 24 h. This study provides valuable insights into the feasibility of using DL for hypertension risk assessment, particularly through PPG recordings. It demonstrates that closely spaced calibration measurements can lead to highly accurate classification, emphasizing the potential for real-time applications. These findings may pave the way for advanced, non-invasive, and continuous blood pressure monitoring methods that are both efficient and reliable.

## 1. Introduction

Hypertension is a primary risk factor for a wide range of cardiovascular diseases (CVD), including stroke, ischemic heart disease, heart failure, and peripheral arterial disease. Research has shown that improving blood pressure (BP) control can prevent more deaths than any other major risk factor modification. Early identification, diagnosis, and treatment of hypertensive individuals are crucial before achieving effective BP control [1]. Regular BP monitoring is essential for the entire population, with special emphasis on individuals previously diagnosed with hypertension, as they are particularly vulnerable to elevated BP. Shockingly, more than 60% of hypertensive patients continue to have uncontrolled hypertension. Out-of-office monitoring offers the advantage of obtaining multiple readings over time, enabling early detection of asymptomatic individuals without acute target organ damage and facilitating hypertension management [2].

For intermittent non-invasive BP measurement, the use of an occluding upper arm cuff is the gold standard due to its high accuracy. BP values are obtained manually through palpation or auscultation of Korotkoff sounds, or automatically through oscillometry with a pressure sensor. Continuous non-invasive BP monitoring can be achieved using techniques such as arterial applanation tonometry or the volume clamp method [3]. However, cuff-based devices are uncomfortable and unsuitable for continuous measurements during daily activities or exercise, as they require the user to keep their arm immobile and possess knowledge of measurement procedures [4].

Recent advancements in digital technology have led to the development of robust wearable BP monitoring sensors. The ideal wearable device should be non-invasive, inconspicuous, compatible with continuous use over periods ranging from minutes to months, lightweight, energy-efficient, and adaptable to various activities and locations [5].

Photoplethysmography (PPG) is an optical measurement technique that has been used to create small, cost-effective sensors for monitoring changes in blood volume within the microvascular bed of tissues. PPG waves provide valuable cardiovascular information for clinical physiological monitoring, including blood oxygen saturation, BP, heart rate variability, and pulse wave velocity [6]. PPG sensors consist of red and infrared light-emitting diodes (LEDs) and a detector. These sensors monitor changes in reflected light intensity, which correspond to blood volume changes in the tissue, thereby providing cardiovascular information [7]. PPG is a promising, cost-effective method with a high correlation with arterial BP in both frequency and time domains [8].

In recent years, artificial intelligence (AI) has been applied to estimate or discriminate between blood pressure levels. On the one hand, machine learning (ML) techniques combine electrocardiography (ECG) and PPG signals, utilizing propagation theory and parameters such as pulse transit time (PTT), pulse arrival time (PAT), and pulse wave velocity (PWV) to assess how BP affects the cardiovascular system [9]. Recent studies combine these propagation parameters with PPG morphological feature extraction, using them as inputs for regression methods [10], support vector machines [11], or neural networks for BP determination [12].

On the other hand, deep learning (DL), a promising subfield of machine learning (ML), has significantly advanced in terms of its utilization and efficiency. These advancements are attributable to the availability of extensive data, the development of neural network models, and the progress in computing hardware, including central processing units (CPUs) and graphics processing units (GPUs) [13]. Currently, DL techniques are considered state-of-the-art for image classification, exemplified by the fact that all the winning entries in the ImageNet Large Scale Visual Recognition Competition utilized deep convolutional neural networks (CNNs) for classification [14].

CNNs are analogous to traditional Artificial Neural Networks (ANNs) in that both employ neurons that receive input and perform non-linear operations to derive a final output representing class scores, achieved through weight vectors. However, CNNs are primarily employed in tasks related to pattern recognition in images, as they are adept at encoding specific features of the images within their architectural framework [15]. The key advantages of CNNs include a reduction in the number of parameters when dealing with large models designed to tackle complex tasks. CNNs eliminate the need for manual feature extraction, making them robust in handling new and diverse images for classification. Additionally, CNNs do not rely on spatially dependent features, enabling them to detect patterns regardless of their location within an image. Another significant advantage is that abstract features are progressively extracted as the input image propagates through deeper layers of the network. Initial layers focus on detecting edges, followed by more complex shapes, and ultimately, higher-level features [16].

For effective training of a CNN, it is crucial to optimize hyperparameters such as the batch size, defined as the number of images used in each training epoch, and the learning rate or step size at each iteration. An algorithm with an excessively small learning rate hyperparameter will converge slowly, while an overly large one may cause the training process to diverge [17]. Moreover, a batch size that is too small can result in the algorithm oscillating without achieving acceptable performance, while excessively high batch sizes can lead to convergence without meaningful improvements in accuracy [18].

As a result, recent studies have employed DL techniques for the classification of blood pressure based on PPG signals, utilizing image representations generated through techniques such as continuous wavelet transform (CWT) [19] and short-time Fourier transform (STFT) [20,21]. Notably, one of the primary advantages over traditional ML methods is that DL eliminates the need for manual feature extraction from PPG signals, as the most relevant features are automatically extracted from the input images, rendering ECG signals unnecessary. This streamlines the practical application of the model in wearable devices.

The incorporation of these algorithms into wearable devices has the potential to provide reliable insights into the blood pressure status of monitored individuals. This contribution can play a pivotal role in the prevention, early diagnosis, and ongoing management of hypertension and related cardiovascular conditions. The growing prevalence of wearable devices, smartphones, and biosensors has ushered in a medical revolution, enabling the integration of AI tools to address complex medical challenges. These technologies are rapidly becoming the cornerstone of vital sign monitoring and are pivotal in achieving optimal diagnoses and treatment follow-up, while empowering patients to take a more active role in their healthcare journey [7].

In this study, we aim to develop a classification system using DL-based methods to discriminate between normotensive (NTS) and hypertensive (HTS) individuals, while evaluating the necessity and effectiveness of per-subject calibration to improve classification results using ML-based approaches. We leverage PPG recordings transformed into scalograms through CWT as input images for pretrained CNNs, eliminating the need for simultaneous ECG recordings or manual feature extraction, allowing the DL models to automatically extract deep features from PPG signals. Furthermore, we demonstrate that DL models performed optimally when calibration and measurements occurred close in time. However, as the time interval between calibration and measurements increased, accuracy gradually decreased. Nonetheless, even with calibration intervals exceeding 24 h, all models maintained an acceptable accuracy above 71%. Finally, our findings suggest that DL models, especially ResNet-18, have the potential to be integrated into wearable devices and digital health solutions for continuous monitoring of blood pressure.

The manuscript is organized as follows. Section 2 presents the database. Section 3 presents the previous ML-based method procedure, actual DL-based method procedure and models evaluation. Section 4 presents the results, that will be analyzed in Section 5. Finally, in Section 6 the main scientific contributions are remarked.

## 2. Materials

The signal dataset used in this study was obtained from the MIMIC, a freely-available database, which contains biomedical recordings from ICU patients admitted to the Beth Israel Deaconess Medical Center in Boston, MA, USA [22]. Although ICU patients recorded in MIMIC are not completely representative of the general population, for this research it has allowed to obtain simultaneous recordings of PPG and invasive BP signals over long periods of time, allowing to study different periods between calibration and blood pressure measurement. After obtaining the recordings, a manual quality check was conducted to identify and eliminate signals with noise or artifacts. PPG and BP signals were collected from commercial devices and a catheter in the radial artery, and significant artifacts could arise due to sensor issues, patient movements, or interference with physiological signals, causing deviations from their characteristic morphology.

In this study, a binary classification approach was adopted to discriminate between NTS and HTS subjects, as the clinical focus was primarily on detecting hypertension over the normotensive state. To achieve this, a systolic blood pressure (SBP) threshold of 130 mmHg was established to distinguish between the two states. It’s worth noting that the report of the Joint National Committee on the prevention, detection, evaluation, and treatment of high blood pressure [23] clinically defines a third category for prehypertensive subjects with SBP values between 120 and 140 mmHg. However, since prehypertensive patients with stable SBP values within this range are relatively uncommon, the primary objective of this study was to develop tools capable of detecting hypertension or its initial symptoms, i.e., when a patient’s SBP exceeds 130 mmHg.

Additionally, subjects displaying significant fluctuations in their SBP values between NTS and HTS measurements over time were excluded from the dataset. Ultimately, a total of 974 recordings from 69 subjects were selected from the MIMIC database, with 45 subjects classified as NTS and 24 as HTS. PPG and ABP signals were recorded simultaneously, with a duration of 120 s, a common sampling frequency of 125 Hz, and a resolution of 8–10 bits [24].

## 3. Methods

In this section, the methodologies employed are outlined, with a particular focus on the utilization of DL techniques in comparison to traditional ML. The ML-based method is initially described, emphasizing the role played by ECG signals and the complex processes of feature definition and selection. Subsequently, the DL-based method is introduced, highlighting the signal preprocessing steps, the application of CNNs, and the necessity of calibration for performance enhancement. The evaluation metrics for classification performance are elucidated, and the experimental setups examining the impact of varying time intervals between calibration and measurement are detailed.

### 3.1. Machine Learning-Based Method

**Figure 1:**
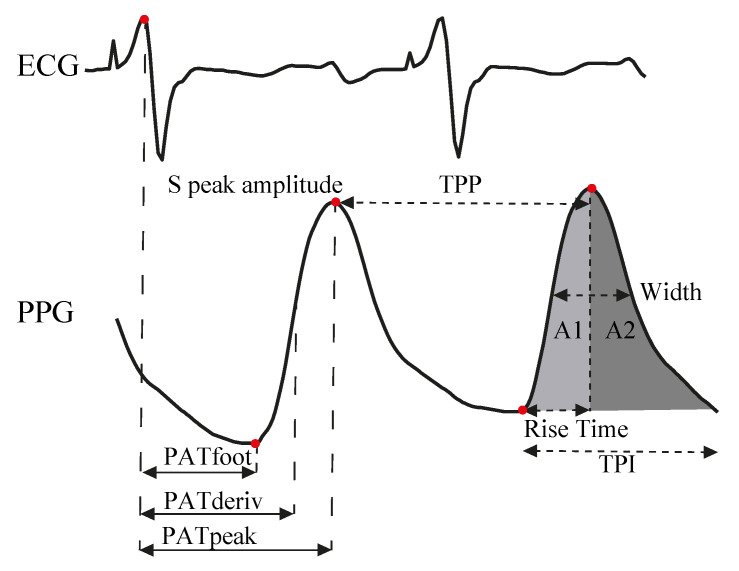
Main discriminatory features defined in machine learning based method: PAT, PPG peak amplitude, time peak to peak (TPP), width, areas under the pulse and time pulse interval (TPI) [25].

As mentioned previously, the primary objective of this study was to evaluate whether the classification of hypertension risk using PPG signals could be improved using DL-based methods as compared to traditional ML-based methods. To provide context, we will summarize the key elements of the ML methodology utilized in a previous work [25]:

- In addition to PPG and ABP signals, the ECG signal is a prerequisite, as it plays a critical role in the extraction of pulse arrival times (PAT), a pivotal discriminant feature. PAT is defined as the time interval between the R-peak in the ECG signal and three fiducial points in the PPG signal [26].
- Fiducial points are identified in the PPG signal, as well as in velocity plethysmogram (VPG) and acceleration plethysmogram (APG) signals, through the application of first and second-order derivatives, respectively.
- The methodology defines 23 discriminatory features based on the pulse wave propagation model and morphological characteristics. Key features are illustrated in Figure 1.
- Feature selection is performed for dimensionality reduction and performance improvement. Features with low correlation [27] and high discrimination [28] were selected The goal is to retain only those features that are most relevant for assessing hypertension risk. This process results in a selection of 17 features, which are used as inputs for ML-based classification models.
- The selected ML classification models include Support Vector Machines (SVM), k-Nearest Neighbors (KNN), and a Bagging Ensemble classifier, as these models demonstrated the highest classification accuracy.

### 3.2. Deep Learning-Based Method

**Figure 2:**
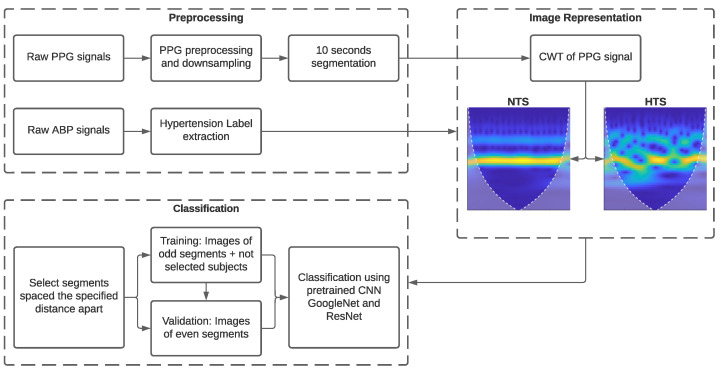
Block diagram illustrating the DL classification methodology, including the study of the need for calibration.

Figure 2 depicts a block diagram illustrating the various stages developed in this research. To begin with, basic signal preprocessing was applied to the PPG signal. Subsequently, the processed signals were transformed into images using CWT. Finally, we examined the necessity for calibration using GoogLeNet, ResNet-18, and ResNet-50 CNNs in conjunction with transfer learning.

#### 3.2.1. Signal Preprocessing

SBP was extracted directly as the mean values of the peaks of each BP waveform since these signals were clear and did not require additional processing. SBP was used to classify subjects into the NTS and HTS groups, with SBP values lower and higher than 130 mmHg, respectively. The PPG signals required the application of a fourth-order Chebyshev II band-pass filter with cutoff frequencies between 0.5 and 10 Hz [29] to eliminate noise and minor artifacts that did not lead to signal rejection in the initial stage of signal quality assessment.

In ML-based methods, a high sampling rate is needed to extract morphological features from the PPG signal. In contrast, with DL-based methods, it is sufficient to preserve the signal waveform, as the most relevant discriminatory features are automatically extracted from the image representation of the PPG signal. Thus, the PPG signals were downsampled from a sampling frequency of 125 Hz to 25 Hz. Using lower sampling rates significantly reduces the amount of data transmitted and saved when signals are acquired and processed with wearable devices [30].

Finally, as the DL method required a large number of images to create an effective classification model, the 120-s recordings of PPG and ABP were divided into 10-s sub-segments. Consequently, the total number of sub-segments analyzed was 11,688.

#### 3.2.2. Pretrained CNN Architecture

For the problem of hypertension risk assessment, pretrained CNNs ResNet and GoogLeNet were employed. The ResNet network employs a residual learning framework to simplify the training of deeper networks. Contrary to what one might expect theoretically, adding more layers to a neural network can lead to saturation in accuracy and rapid degradation due to the degradation problem. The proposed solution to this issue, and one of its main advantages, is residual learning, which involves adding the input of the hidden layers to their output, allowing the network to learn only the residual mapping.

Comparing ResNet-18 with ResNet-50, ResNet-18 is simpler and, therefore, computationally more efficient, making it suitable for scenarios with limited computational resources. However, this simplicity comes at the cost of not being able to capture more complex features, affecting the final accuracy. On the other hand, ResNet-50 achieves superior results owing to its depth and skip connections, but it requires higher computational expenses and is prone to overfitting [31].

GoogLeNet introduced an architecture for computer vision known as “Inception”, which attempts to select the appropriate kernel size for convolutional operations, neural network size, and computational resources. Different parts of an image may require different kernel sizes to extract information efficiently. Moreover, increasing the size and depth of a neural network can lead to overfitting, increased consumption of computational resources, and inefficiency. To address these challenges, Inception modules create a “wider” network rather than a “deeper” one. Other GoogleNet advantages are the capability to capture complex features and the computational efficiency. Nevertheless, it can be time consuming due to its depth [32].

As CNNs require a large amount of data to be trained from scratch, transfer learning methods leverage background knowledge obtained after training a base network and transfer it to solve other relevant problems [33]. Therefore, ResNet and GoogLeNet models can automatically extract deep, powerful, and informative features without the need for manual morphological feature extraction. Furthermore, these models were originally trained to recognize more than 1000 objects and can be retrained with a new set of images by fine-tuning the existing weights and layers much faster and easier than starting from scratch [34]. In any case, it is important to highlight that the focus of our study was not to introduce novel DL architectures but to demonstrate the effectiveness of image-based representations derived from PPG signals in hypertension risk assessment.

**Figure 3:**
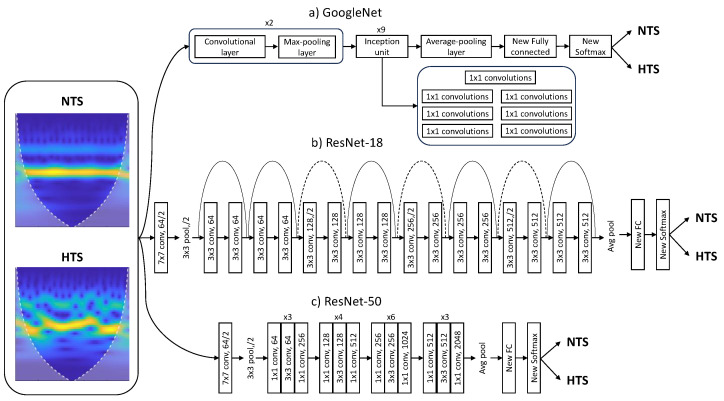
Block diagram illustrating the modified architectures of (**a**) GoogleNet, (**b**) ResNet-18, and (**c**) ResNet-50. To align these models with the new task of assessing hypertension risk using PPG recordings, the original fully connected, softmax, and classification layers have been replaced. Additionally, input images with both labels have been resized to 224 × 224 × 3 to meet the specific requirements of the studied models.

To retrain the pretrained networks, the last three layers were replaced to adapt the model to the new image dataset. These layers use image features and information from the previous convolutional layers to classify the input images. The original fully connected layers were replaced with new fully connected layers, with the same number of outputs as the number of classes, in this case, 2 (NTS or HTS). Similarly, the original softmax layer and the original classification layer were replaced with two new layers of the same type. Figure 3 illustrates the block diagram of the modified architectures of Googlenet and ResNet CNNs.

The Adam optimizer was used in the CNN models for classification with an initial learning rate of $1\times 10^{-4}$, a minimum batch size of 128, and a maximum of 20 epochs. The validation frequency was adjusted based on the number of training images and the ratio between training images and the minimum batch size.

In supervised learning, overfitting can be a significant issue when the model struggles to generalize from the training set to the testing set. This issue was mitigated in this work through the use of early stopping, a technique that automatically halts training when the validation error starts to increase [35].

#### 3.2.3. Image Representation of PPG Signal Using CWT

Since both ResNet and GoogLeNet CNNs require inputs of size 224 × 224 × 3 in Red-Green-Blue (RGB) format, PPG segments were processed using CWT [36] and transformed into a scalogram, which provided the absolute value of CWT coefficients of the PPG signal. These scalograms were then resized to 224 × 224 × 3 to be fed into the training models. The absolute value of the wavelet coefficients was obtained using the analytic Morse (3, 60) wavelet with the Voices per Octave set to 12. The choice of CWT and scalograms was based on their effectiveness in providing time-frequency information and useful learning features for CNNs [19].

Additionally, white lines were added to the scalogram to mark the cone of influence. This marks areas in the scalogram that may be affected by border distortions due to missing values at the beginning and end of the transform. The unshaded region guarantees that the information is an accurate time-frequency representation of the PPG signal.

### 3.3. Classification Model Evaluation

Statistical tests for accuracy (*Acc*), sensitivity (*Se*), specificity (*Sp*), and *F1-Score* were employed for evaluating the classification performance. *Acc* represents the percentage of correctly classified PPG segments. *Se* and *Sp* denote the ability to detect HTS subjects as positive and the ability to detect NTS healthy subjects as negative, respectively. *F1-Score* is the harmonic mean of *Se* and *Acc*. These statistical tests were mathematically computed as follows:

(1) $$Acc=\frac{TP+TN}{TP+TN+FP+FN}$$

(2) $$Se=\frac{TP}{TP+FN}$$

(3) $$Sp=\frac{TN}{TN+FP}$$

(4) $$F1\mathrm{-Score}=\frac{2\cdot Se\cdot Acc}{Se+Acc}=\frac{2\cdot TP}{2\cdot TP+FP+FN}$$

Here, TN represents the number of correctly classified NTS segments, TP is the number of correctly classified HTS segments, FN is the number of incorrectly predicted HTS segments, and FP is the number of incorrectly predicted NTS segments.

### 3.4. Experiment

In our previous research [25], we investigated the importance of calibration in hypertension risk assessment using ML methods, aiming to improve the initial accuracy of 51.48%. This initial accuracy was achieved when a new subject entered the method without any prior per-subject calibration. To study the effect of calibration, we conducted experiments where calibration measurements were taken at various time intervals from the test measurements. In the calibration approach with closely spaced measurements, we performed sequential validation with the twelve sub-segments into which each 120-s segment was divided. This approach was not feasible with DL-based methods due to their high computational complexity and long training times. Consequently, the model would require more training time than the time difference between calibration and test measurements. Furthermore, it would not be practical to perform a calibration and predict hypertension risk only a few seconds later, as the image transformations of both measurements would be very similar.

In this study, we examined the effectiveness of calibration with different time intervals between PPG segments of different subjects. The intervals included less than 1 h, between 1 h and 6 h, between 6 h and 24 h, and more than 24 h apart. For this purpose, we trained CNN classification models following the procedure outlined below:

1. Selection of 120-s PPG segments obtained for each time interval between measurements.
2. The images of odd segments (1st segment, 3rd segment, etc., spaced at each interval) were used for training, and the images of even segments were used for validation. Additionally, images corresponding to PPG signals from subjects who did not have segments separated by the specified time interval were added to the training dataset. This increased the training dataset with subject segments that were not part of the validation dataset, enhancing the model’s robustness.
  In this way, the calibration stage was simulated using training segments, with validation segments between two calibration measurements for classification.
3. These images were used as inputs for GoogLeNet, ResNet-18, and ResNet-50 pretrained CNN models, and the classification problem involved discriminating between normotensive and hypertensive subjects using PPG signal image representations.
4. Classification performance was evaluated using the statistical tests described in Section 3.3. This procedure was repeated for each measurement interval.

Table 1 displays the number of images used in the training and validation tasks for each segment interval. For measurement intervals less than 24 h, the number of images from subjects with segments spaced at these intervals was higher than from unselected subjects. However, the opposite is observed when the interval between measurements exceeds 24 h, as the number of subjects with PPG recordings of more than 1 day is limited, and most of them do not belong to this group.

## 4. Results

The training performance, in terms of the number of epochs after early stopping, training time, and statistical results of classification performance achieved by the proposed CNNs for discriminating between NTS and HTS segments, is presented in Table 2. Although the maximum number of epochs was set to 20, no model reached this limit, indicating the effectiveness of the early stopping technique in preventing overtraining.

All models achieved their highest performance when calibration and measurements were separated by less than 1 h, resulting in accuracy exceeding 90%. Among them, ResNet-18 exhibited the best classification results, achieving an accuracy of 93.32%, sensitivity of 84.09%, specificity of 97.30%, and an F1-score of 88.36%. Furthermore, this model demonstrated efficiency, requiring only 6 epochs and a training time of 28 min. As expected, the classification performance decreased as the time interval between calibration and test measurements increased, with accuracy falling below 81% for distances above 6 h.

## 5. Discussion

Hypertension is a major public health concern worldwide, with a high prevalence and significant morbidity and mortality rates. Early identification and treatment of hypertensive individuals can prevent deaths and reduce the risk of cardiovascular diseases. Wearable devices, smartphones, and biosensors have ushered in a medical revolution, enabling the integration of AI tools to address complex medical challenges. These technologies are rapidly becoming the cornerstone of vital sign monitoring and are pivotal in achieving optimal diagnoses and treatment follow-up, while empowering patients to take a more active role in their healthcare journey [37].

Our study leverages a dataset from the MIMIC database, consisting of PPG recordings and invasive blood pressure (BP) measurements. We employed deep learning (DL) models, specifically GoogLeNet, ResNet-18, and ResNet-50, for hypertension risk discrimination. Compared to previous works in the field, our methodology incorporates the use of CWT-based scalograms, which allow us to automatically extract deep features from PPG signals without the need for manual feature extraction [9,38]. This marks a departure from traditional machine learning-based approaches that rely on handcrafted features from PPG signals and ECG data.

The models were evaluated under four different calibration intervals: below 1 h, between 1 and 6 h, between 6 and 24 h, and above 24 h. The results show that the models achieved their highest accuracy when the calibration and measurements were separated by less than 1 h. In this scenario, ResNet-18 exhibited the best classification results, achieving an accuracy of 93.32%, sensitivity of 84.09%, specificity of 97.30%, and an F1-score of 88.36%. This indicates that close calibration intervals improve classification outcomes significantly. As the time interval between calibration and test measurements increased, the classification performance decreased. For intervals above 6 h, the accuracy fell below 81%. In any case, all models maintained accuracy above 71% even for intervals above 24 h. It is important to note that the early stopping technique was effective in preventing overtraining, as no model reached the maximum set number of epochs (20). The training times varied between models, with ResNet-50 having the longest training time.

This suggests that DL-based models, particularly ResNet-18, hold promise for accurate hypertension risk discrimination from PPG signals. Consequently, this technology can be integrated into wearable devices and digital health solutions for continuous monitoring.

Prior research in this field has explored the application of DL models for hypertension risk stratification by employing image representations of PPG signals, such as the Hilbert–Huang Transform [39] and CWT [19,40], achieving F1-Scores exceeding 92%. However, these studies did not adhere to a rigorous classification procedure, as they performed signal segmentation before dividing the dataset into training and validation sets. Consequently, images representing consecutive signal segments were included in both the training and validation subsets, potentially leading to an overestimation of the reported results. The calibration method proposed in this study significantly enhances these strategies, providing a classification approach that aligns with the decision-making process of a model that can be easily integrated into wearable devices.

In contrast to our earlier research [25], where we investigated the significance of calibration in ML-based hypertension risk assessment, and taking into account that the methodology employed in this study differs from that detailed in Section 3.1 and Section 3.4, we have observed remarkably consistent accuracy results across the four intervals between calibration and measurement. Notably, the DL-based method brings forth a significant improvement by eliminating the requirement for ECG signals, with features being automatically extracted from the images.

Despite the numerous benefits of AI, it is essential to consider the limitations and challenges associated with its application in the context of vital sign monitoring and medical diagnosis. Moral dilemmas and communication barriers between physicians and patients may arise. Furthermore, privacy issues related to sensitive health data, the potential for re-identification through AI processes, and concerns about data breaches are prominent. The ethical discourse surrounding AI in healthcare, where large datasets are required, should address these privacy concerns to ensure responsible and secure implementation [41]. To this respect, the use of DL models entails the processing of sensitive health data, including PPG signals and blood pressure measurements. Ensuring the confidentiality and security of this information is paramount to maintain patient trust and comply with data protection regulations. Striking a balance between leveraging the potential benefits of DL for medical diagnosis and safeguarding patient privacy remains a critical challenge [42].

Moreover, the interpretability of DL models poses ethical challenges. The inherent complexity of neural networks makes it challenging to provide clear explanations for the decisions made by these models. In a healthcare setting, where transparency and interpretability are crucial, addressing the “black box” nature of DL algorithms becomes imperative [43]. Understanding how these models arrive at specific hypertension risk assessments is essential for physicians, patients, and regulatory bodies.

As we consider the integration of DL into wearable devices for real-time monitoring, additional ethical considerations come to the forefront. Wearables offer the potential for continuous, unobtrusive monitoring of individuals’ cardiovascular health. This continuous monitoring, while advantageous for early detection and intervention, raises concerns about the continuous surveillance of individuals’ health data. Striking a balance between the benefits of proactive healthcare and the right to privacy is a delicate ethical challenge. Furthermore, while wearables offer the promise of timely health insights, issues such as device accuracy, user adherence, and data transmission security need careful consideration [44,45]. Ensuring that wearable devices are reliable and provide accurate hypertension risk assessments is crucial for their successful integration into routine healthcare practices.

Other limitations commonly encountered when applying DL in the medical field include the requirement for large datasets to attain optimal performance, challenges related to overfitting where the model trained on data may struggle to generalize effectively with new, unseen data, the computational demands associated with DL models, issues arising from imbalanced datasets where there is a significant disparity between positive and negative samples, and the interpretability concern. In the medical context, interpretability is crucial as it necessitates not only predicting outcomes but also understanding and explaining how the model arrives at its decisions [46].

The study presents a significant contribution to the field of hypertension risk discrimination, demonstrating the potential of DL and calibration methods to improve the accuracy of hypertension risk discrimination. The incorporation of these methods into wearable devices has the potential to provide reliable insights into the blood pressure status of monitored individuals, playing a pivotal role in the prevention, early diagnosis, and ongoing management of hypertension and related cardiovascular conditions. This study highlights the importance of individualized calibration for accurate hypertension risk discrimination, demonstrating the potential of this approach to achieve optimal diagnoses and treatment follow-up, while empowering patients to take a more active role in their self healthcare.

## 6. Conclusions

This study leveraged DL models combined with proper calibration to assess hypertension risk using PPG signals where the use of CWT-based scalograms eliminated the need for manual feature extraction. The models performed best when calibration and measurements were close to each other and accuracy decreased as the time interval between calibration and test measurements increased. Furthermore, the present study emphasizes the significance of individualized calibration for accurate hypertension risk assessment. Finally, the future research plan involves attempting to implement these methods into wearable devices to improve early diagnosis and management of hypertension and related cardiovascular conditions.

## Figures and Tables

**Table 1:** Division of PPG signals’ image representation in training and validation datasets for each measurement interval.

	Training Images		Validation Images
	Odd Segments	Not Selected Subjects	Even Segments
Below 1 h	2136	408	1752
Between 1 h and 6 h	3168	84	2784
Between 6 h and 24 h	1728	732	1356
Above 24 h	288	8868	192

**Table 2:** Classification performance for distinguishing between NTS and HTS individuals using GoogLeNet, ResNet-18, and ResNet-50 pretrained CNNs with calibration distances below 1 h, between 1 and 6 h, between 6 and 24 h, and above 24 h.

Model	Epochs	Training Time	Accuracy	Sensitivity	Specificity	F1-Score
**Below 1 h**						
GoogLeNet	13	64 min	90.18%	82.77%	93.38%	83.56%
ResNet-18	6	28 min	93.32%	84.09%	97.30%	88.36%
ResNet-50	8	115 min	92.52%	84.09%	96.16%	87.14%
**Between 1 h and 6 h**						
GoogLeNet	9	61 min	82.87%	73.12%	88.40%	75.55%
ResNet-18	15	107 min	89.04%	81.15%	93.52%	84.29%
ResNet-50	8	152 min	88.11%	80.16%	92.62%	83.00%
**Between 6 h and 24 h**						
GoogLeNet	14	66 min	79.57%	70.57%	85.98%	74.18%
ResNet-18	6	29 min	80.16%	73.23%	85.10%	75.43%
ResNet-50	19	99 min	80.68%	76.60%	83.59%	76.73%
**Above 24 h**						
GoogLeNet	7	57 min	73.37%	38.73%	93.68%	51.80%
ResNet-18	7	32 min	79.17%	68.14%	85.63%	70.74%
ResNet-50	19	221 min	71.56%	56.86%	80.17%	59.64%

## Data Availability

The data supporting reported results and presented in this study are available on request from the corresponding author.
